# Effect of* Lonicera caerulea* var.* emphyllocalyx* Extracts on Murine* Streptococcus pyogenes* Infection by Modulating Immune System

**DOI:** 10.1155/2019/1797930

**Published:** 2019-02-07

**Authors:** Masaaki Minami, Mineo Nakamura, Toshiaki Makino

**Affiliations:** ^1^Department of Bacteriology, Graduate School of Medical Sciences, Nagoya City University, 1 Kawsumi, Mizuho-ku, Nagoya, Japan; ^2^Nakamura Pharmacy, 7- North5-1 Nango-Dori, Shiraishi-ku, Sapporo, Japan; ^3^Department of Pharmacognosy, Graduate School of Pharmaceutical Sciences, Nagoya City University, 3-1 Tanabe-Dori, Mizuho-ku, Nagoya, Japan

## Abstract

*Streptococcus pyogenes* (*S. pyogenes*) causes several infectious diseases such as tonsillitis, cellulitis, and streptococcal toxic shock syndrome. The general treatment of* S. pyogenes* infection is by using *β*-lactam antibiotics; however, the cases of treatment failure were increasing as serious problems.* Lonicera caerulea *var.* emphyllocalyx* (LCE) has been used in the folk medicine in the northern part of Japan, the northern part of China, Korea, and Russia. In this study, we investigated the efficacy of three parts (fruit, stem, and leaf) of* Lonicera caerulea *var*. emphyllocalyx *extract (LCEEs) against murine* S. pyogenes* infection. Oral administration of LCEEs increased the mortality in murine model, and the extracts of its stems and leaves were more effective than the fruit extract significantly. Murine splenocytes and mesenteric lymph nodal cells treated with LCEEs suppressed the excess production of inflammatory cytokine such as TNF-*α* in comparison to those from untreated cells. LCEEs stimulated the differentiation of pluripotent hematopoietic stem cells in those murine lymph nodal cells. It also activated the proliferative response of murine lymph nodal cells. We also found that the stem and leaf extracts seemed to be more effective than the fruit extract in those phenomena. The concentration of lignins in LCEE prepared from the stems was larger than that from leaves, and that was larger than that from the fruits. Our data suggest that LCE, especially the stems and the leaves, may be useful for the treatment of* S. pyogenes* infection.

## 1. Introduction

*Streptococcus pyogenes *(*S. pyogenes*) is a gram-positive pathogenic bacterium. Because it has several virulent factors such as streptolysin O, streptolysin S, NADase, SpeB protease, and streptococcus inhibitory of complement lysis, it causes various infectious diseases such as pharyngitis, tonsillitis, nephritis, cellulitis, and necrotizing fasciitis [1]. As the drug such as macrolide and tetracycline, resistant rate of* S. pyogenes* is gradually increasing worldwide including Japan [2]; novel anti-*S. pyogenes* drug besides popular antibiotics has been desired.

*Lonicera caerulea *var*. emphyllocalyx* (LCE) belongs to honeysuckle family (Caprifoliaceae) and* Lonicera* genus, which is known as edible berries [3]. LCE lives in the northern temperate zone such as the northern part of Japan (Hokkaido), the northern part of China, Korea, and Russia. It is currently commercially produced in Japan and Russia [3]. The fruits, flowers, leaves branches, and bark of honeysuckle plants were used in the folk medicine in the countries of their origin. For example, branch infusion has been used as a diuretic remedy. As fresh fruit juice has been used as a general strengthening means, they were also recommended for the treatment of some disease of the stomach and tonsillitis for antiseptic effect [4]. Although this mode of action has not been unclear, in recent years, a large number of studies have investigated the therapeutic effects of berries in the prevention of a range of diseases and there is in increasing interest in herbal products [3]. Berries constitute the several important sources of potential health supporting phytochemicals in the human diet [5]. They contain carbohydrates, lipids, and proteins, organic acids and also ascorbic acid, Vitamin B, magnesium, phosphorus, calcium, and potassium as minor compounds [6, 7]. They have antitumorigenic, antimicrobial, anti-inflammatory, and antimutagenic properties [8–11].

Among the herbal medicines used in Japanese traditional medicine (Kampo medicine) and traditional Chinese medicine, the flower bud, stems, and leaves of* Lonicera japonica* are formulated into a prescription with indications such as the febrile common cold, influenzae infection, and the joint pain [12–14]. However, the scientific report of stem and leaf from LCE about health science had been seldom known.

The gut-associated lymphoreticular tissues (GALT) including mesenteric lymph nodes exist on the intestinal mucosal sites and play an important role in host defence including IgA response in the mucosal immune system [15]. The cytokine network also plays an important role in the inflammatory and immune responses in total immune system [16]. As LCE are taken orally, the digestive mucosal immune system including mesenteric lymph nodes may act as one of the major targets for the expression of pharmacological activity. However, the modulating activity of LCE on GALT system has not been unclear.

Therefore, we hypothesized that LCE which is one of* Lonicera* genus may have anti-infective activity through GALT system. In this study, we tried to clarify whether LCE is novel candidate for anti* S. pyogenes* therapy. Furthermore, we focus on not only the fruits of, which are edible, but also the stems and leaves for exploration of novel drug. In the present study, we compared the anti-inflammatory effects of LCE by the immunostimulatory effects in the total and local immune system by the induction of granulocyte-macrophage colony-stimulating factor (GM-CSF) secretion from murine splenocytes and mesenteric lymph nodes using several parts of LCE.

## 2. Materials and Methods

### 2.1. Preparation of Samples

LCE was harvested in the field located in Atsuma-Town, Hokkaido, northern part of Japan. LCE fruit is neither an herbal medicine nor a crude drug. People usually take this fruit as fresh one. Although its leaves and stems are not usually taken, some kind of leaves and stems from plant such as* Lonicera japonica* are used as dried herbal products [12–14]. Now we applied this concept for LCE. Therefore, we used fresh fruit and dried leaves and stems as samples. Thus, the 633 g of the fresh fruit, 5.6 g of the leaves, and 20.9 g of the branches (stems) (fresh weight of fruits, dried weight of leaves, and stems) were soaked in 2 L, 500 mL, and 500 mL of MeOH, respectively, and stood for 72 hours at room temperature. After filtration through filter paper, the same amount of MeOH was again added to the residue, and the mixture was allowed to stand for 72 hours at room temperature. After filtration, each filtrate was evaporated under reduced pressure and finally lyophilized. The weights of the extracts (LCEEs) after lyophilization were 79.8 g for fruits, 1.33 g for leaves, and 0.686 g for stems, respectively. The extraction efficiencies were 12.6% for the fruits, 23.8% for the leaves, and 3.28% for the stems, respectively. Fruits, leaves and stem extracts were dissolved and suspended at 200 mg/mL in water, 40% DMSO, and 20% DMSO, respectively, and stored at –20°C.

### 2.2. Evaluation of Animal Challenge Assay

**Figure 1:**
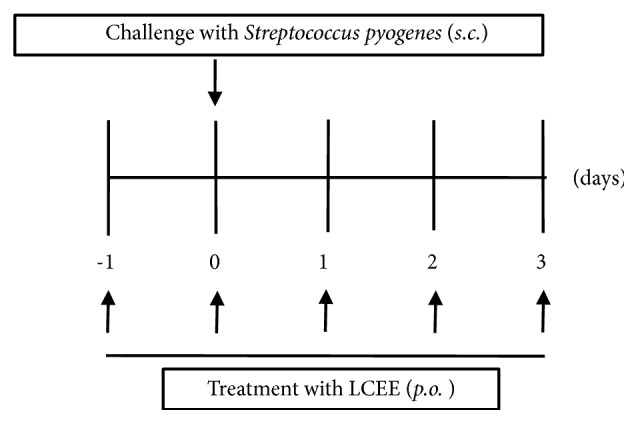
Protocols for the experiments of* S. pyogenes*-induced murine model. In infected groups, 1 x 10^6^ CFU bacteria were injected subcutaneously using a 27-gauge needle at day 0. In the LCEE-treated groups, mice were administrated with each LCEE prepared from fruits, leaves, or stems (1 g/kg body weight/day) orally.

The ability of* S. pyogenes* to cause cellulitis in mice after subcutaneous inoculation was assessed using a procedure described elsewhere [17]. In brief,* S. pyogenes* 1529, which was clinical isolates from severe invasive disease in Japan [17], was harvested after 16-hour growth on brain heart infusion agar (Eiken Chemical, Tokyo, Japan) containing 0.3% yeast extract (BHY agar) mixed in 1 mL of phosphate buffered saline (0.15 M, pH 7.2, PBS) and then centrifuged at 2,000 ×* g* for 2 min. The pellets were diluted in 1 ml PBS to 1×10^8^ CFU and then injected 1 × 10^6^ CFU under the skin surface of inbred 3-week-old female Slc:ICR mice (Japan SLC, Shizuoka, Japan) using a 27-gauge needle. The number of CFU injected was verified for each experiment by plating the bacteria on BHY agar and counting CFU. The general status of mice was observed daily. In the LCEEs-treated groups, mice were gavaged with each LCEE of the fruits, leaves, or stems (1 g/kg/day) on days −1, 0, 1, and 2 after* S. pyogenes* inoculation, respectively. Mice in the control group were given an equal volume of PBS and were infected using the same method (Figure 1). The experimental procedures were conducted according to Nagoya City University Guidelines for the Care and Use of Laboratory Animals, and the study protocol was approved by the local Animal Ethics Committee of Nagoya City University (H24-M11).

### 2.3. Evaluation of Anti-Inflammatory Action in Splenocytes and Mesenteric Lymph Nodal Cells In Vitro

Evaluation of anti-inflammatory action in spleen and mesenteric lymph nodes was performed as described elsewhere [18]. Briefly, splenocytes and mesenteric lymph nodal cells prepared from ICR mice (3-week-old, female) were treated with 2% fatal bovine serum (FBS, Sigma-Aldrich, St. Louis, MO, USA), 100 U/mL penicillin (Wako Pure Chemical, Osaka, Japan), 100 *μ*g/mL streptomycin (Wako), and 10 *μ*g/mL of lipopolysaccharide (LPS) from* E. coli* serotype 026: B6, (Sigma-Aldrich) and LCEEs for 24 hours at 37°C, cultured at 5% CO_2_, and the concentrations of tumor necrosis factor- (TNF-) *α* and interferon- (INF-) *γ* in the subsequent culture medium were measured with ELISA kits (BioLegend, San Diego, CA, USA).

### 2.4. Determination of Proliferative Response of Splenocytes and Mesenteric Lymph Nodal Cells

Determination of proliferative response of spleen and mesenteric lymph nodal cells was described elsewhere [18]. After the mice were sacrificed by CO_2_ inhalation, spleen and mesenteric lymph nodes were removed aseptically and those tissues were filtered and cultured in RPMI1640 medium (Wako) with 5% FBS. The 10 *μ*g/mL of LPS from* E. coli *serotype 026:B6 was added according study. At 20 hours before the end of the splenocyte culture, ^3^H-thymidine (2.0 Ci/mmol; PerkinElmer, MA, USA) was added to the medium. When the culture was finished, the cells were adsorbed on 0.45 *μ*m membrane filters (Advantec Japan, Tokyo, Japan), washed with distilled water, and then dried. The filters were transferred to vials filled with liquid scintillator cocktail, and the radioactivity was measured with a liquid scintillation counter (LSC-6100, Hitachi Aloka Medical, Tokyo, Japan). Results are given as DPM (Disintegration per minute).

### 2.5. Evaluation of Differentiation of Pluripotent Hematopoietic Stem Cells in Splenocytes and Mesenteric Lymph Nodal Cells In Vitro

Evaluation of differentiation of pluripotent hematopoietic stem cells in spleen and mesenteric lymph nodes were performed by modified assay [19, 20]. Splenocytes and mesenteric lymph nodal cells prepared from ICR mice (3 weeks old, female) were treated with 2% FBS, 100 U/mL penicillin, 100 *μ*g/mL streptomycin, and LCEEs for 24 hours at 37°C, cultured at 5% CO_2_, and the concentration of GM-CSF in the subsequent culture medium was measured with an ELISA kit (BioLegend).

### 2.6. Measurement of Lignins

The concentrations of lignins in LCEEs were performed by sulfuric acid method [21]. After the LCEEs (5 mg sample) were taken in beakers, the 72% of sulfuric acid was added, and the mixtures were stirred and allowed to stand at room temperature for 4 hours. The contents of the beakers were transferred to flasks containing distilled water and covered with aluminum foil. This was heated in a high-pressure steam sterilizer at 121°C and 0.08 MPa for 2 hours. At this point, the carbohydrate in the sample was hydrolyzed. After cooling, the black precipitates in the flasks were suction-filtered using a glass filter. The recovered precipitates were washed with hot water, washed with cold water, dried in a dryer at 105°C, and cooled in a desiccator and weighed. As acid soluble lignins, the filtrates were diluted 10 times with 3% sulfuric acid, and the optical densities (340 nm) were measured.

### 2.7. Statistical Analysis

The statistical analysis was conducted using Bonferroni-Dunnett's multiple comparison* t*-test for the differences among multiple groups. Survival data were assessed by Kaplan–Meier survival analysis and tested for significance using the log-rank test.* P*-values less than 0.01 were considered statistically significant (EZR version 1.36).

## 3. Results

### 3.1. Evaluation of Animal Challenge In Vivo

**Figure 2:**
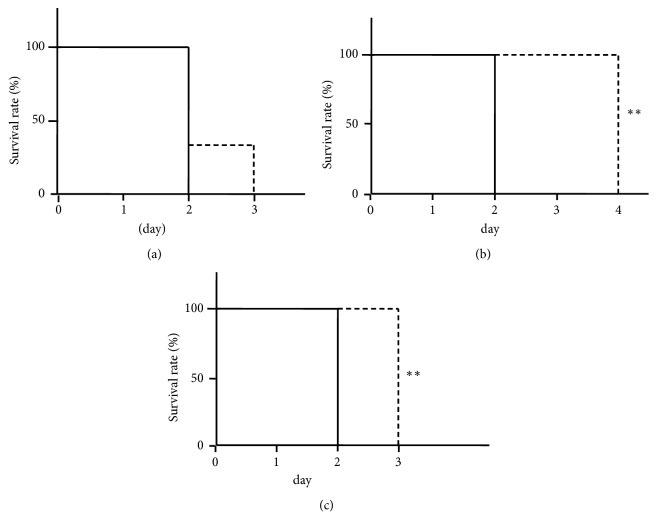
Administration of LCEEs increased the survival rate of* S. pyogenes*-infected murine models. Three-week-old ICR mice were gavaged with LCEEs ((a) fruits, (b) stems, and (c) leaves) for 4 consecutive days (day −1, 0, 1, and 2) and inoculated with 1×10^8^ CFU of* S. pyogenes* 1529 at day 0. Mortality was monitored for 7 days. Survival data were assessed by Kaplan–Meier survival analysis and tested for significance using the log-rank test (*n* = 6). *∗∗p*< 0.01 by Kaplan–Meier survival analysis.

ICR mice were infected subcutaneously with* S. pyogenes* 1529 strain. The following survival curves were monitored for 3 days while orally administering LCEEs. No significant difference of survival rate was observed in the fruit LCEE group (Figure 2(a)). However, the groups treated with LCEEs of leaves and stems observed significantly extended survival rate (Figures 2(b) and 2(c)).

### 3.2. Evaluation of Anti-Inflammatory Action in Splenocytes and Mesenteric Lymph Nodal Cells In Vitro

**Figure 3:**
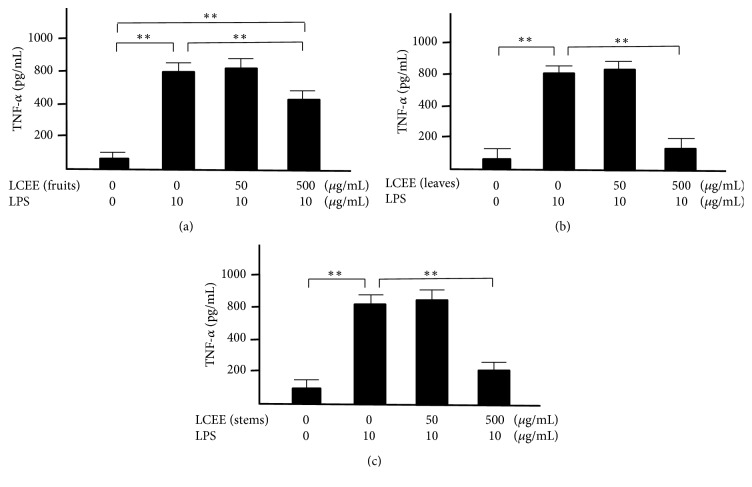
TNF-*α* levels in culture medium of splenocytes. LCEEs ((a) fruits, (b) stems, and (c) leaves) were added to cells. TNF-*α* was measured by ELISA. Data represent the mean ± SD (*n* = 6). *∗∗p*< 0.01 by Bonferroni-Dunnett's* t*-test.

**Figure 4:**
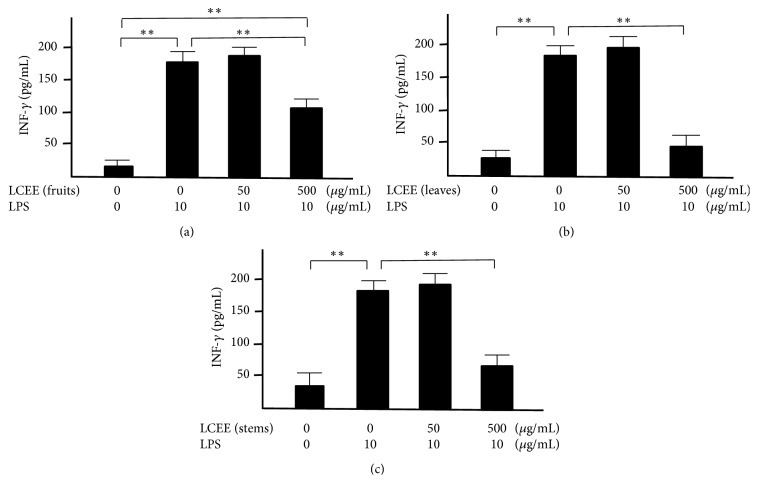
INF-*γ* levels in culture medium of splenocytes. LCEEs ((a) fruits, (b) stems, and (c) leaves) were added to cells. INF-*γ* was measured by ELISA. Data represent the mean ± SD (*n* = 6). *∗∗p*< 0.01 by Bonferroni-Dunnett's* t*-test.

**Figure 5:**
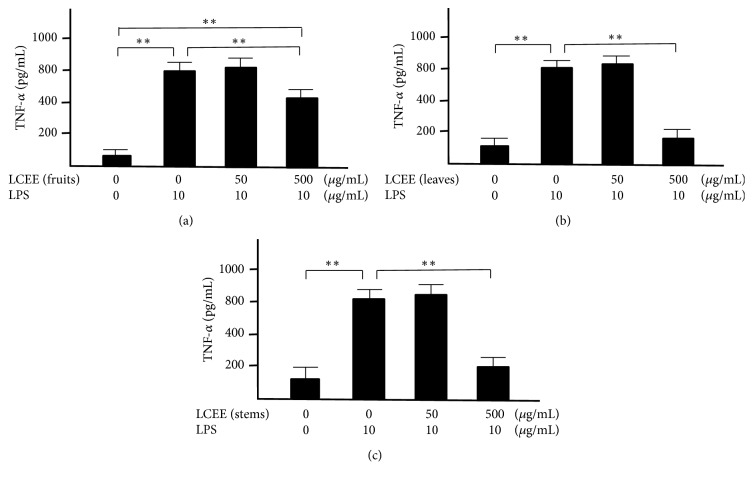
TNF-*α* levels in the culture medium of mesenteric lymph nodal cells. LCEEs ((a) fruits, (b) stems, and (c) leaves) were added to cells. TNF-*α* was measured by ELISA. Data represent the mean ± SD (*n* = 6). *∗∗p*< 0.01 by Bonferroni-Dunnett's* t*-test.

**Figure 6:**
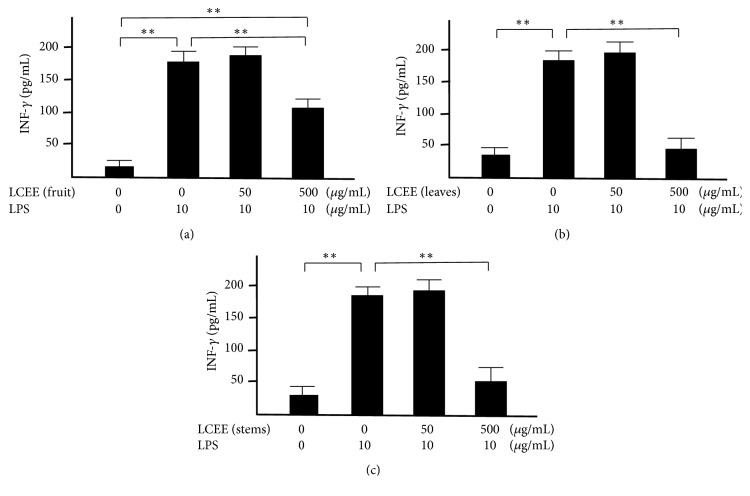
INF-*γ* levels in the culture medium of mesenteric lymph nodal cells. LCEEs ((a) fruits, (b) stems, and (c) leaves) were added to cells. INF-*γ* was measured by ELISA. Data represent the mean ± SD (*n* = 6). *∗∗p*< 0.01 by Bonferroni-Dunnett's* t*-test.

Splenocytes and mesenteric lymph nodal cells isolated from ICR mice were incubated with both LPS and LCEEs from fruits, leaves, and stems for 24 hours, respectively. The concentrations of TNF-*α* and INF-*γ* in the culture medium were measured. LPS significantly induced the productions of TNF-*α* and INF-*γ* in splenocytes and mesenteric lymph nodal cells. And each of LCEEs showed a significant anti-inflammatory effect at a concentration of 500 *μ*g/mL in both splenocytes (Figures 3 and 4) and mesenteric lymph nodal cells (Figures 5 and 6), and the inhibitory activities of LCEEs prepared from the leaves and stems seemed to be significant higher than that of LCEE from the fruits.

### 3.3. Evaluation of Proliferative Response of Splenocytes and Mesenteric Lymph Nodal Cells with LPS

**Figure 7:**
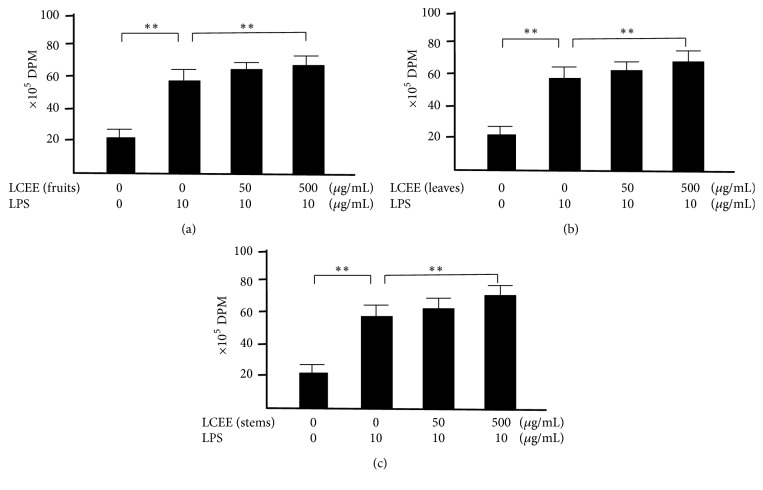
^3^H-thymidine-uptake assay in splenocytes with LPS stimulation. LCEEs ((a) fruits, (b) stems, and (c) leaves) were added to cells. Data represent the mean ± SD (*n* = 6). *∗∗p*< 0.01 by Bonferroni-Dunnett's* t*-test.

**Figure 8:**
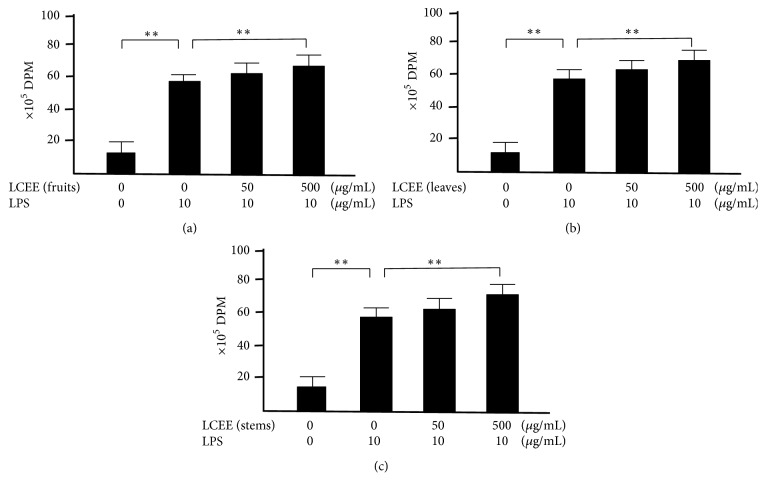
^3^H-thymidine-uptake assay in mesenteric lymph nodal cells with LPS stimulation. LCEEs ((a) fruits, (b) stems, and (c) leaves) were added to cells. Data represent the mean ± SD (*n* = 6). *∗∗p*< 0.01 by Bonferroni-Dunnett's* t*-test.

We focused on the activity of splenocytes and mesenteric lymph nodal cells, because these cells play one of major roles in immune system. To determine whether splenocytes treated with LCEEs showed elevated activities, we performed ^3^H-thymidine uptake analysis. As shown in Figure 7, the uptakes of ^3^H-thymidine into splenocytes treated with LPS were significantly higher than the group without the treatment, and the groups treated with LCEEs (500 *μ*g/mL) were significantly higher than that of untreated groups. We also confirmed the similar results in mesenteric lymph nodal cells treated with LPS and LCEEs. LCEEs (500 *μ*g/mL) significantly induced the proliferation than that of the group treated with LPS (Figure 8).

### 3.4. Evaluation of Differentiation of Pluripotent Hematopoietic Stem Cells in Splenocytes and Mesenteric Lymph Nodal Cells In Vitro

**Figure 9:**
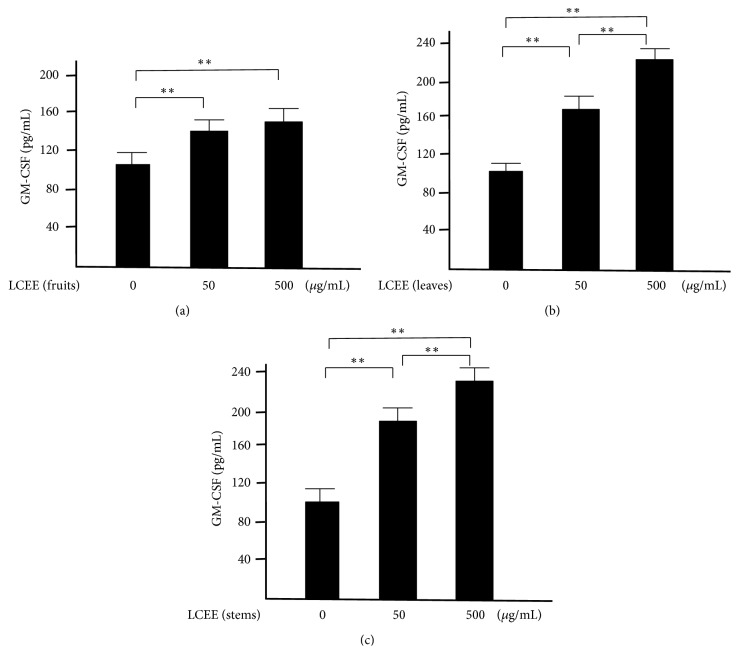
GM-CSF levels in culture medium of splenocytes lymph nodal cells. LCEEs ((a) fruits, (b) stems, and (c) leaves) were added to cells. GM-CSF was measured by ELISA. Data represent the mean ± SD (*n* = 6). *∗∗p*< 0.01 by Bonferroni-Dunnett's* t*-test.

**Figure 10:**
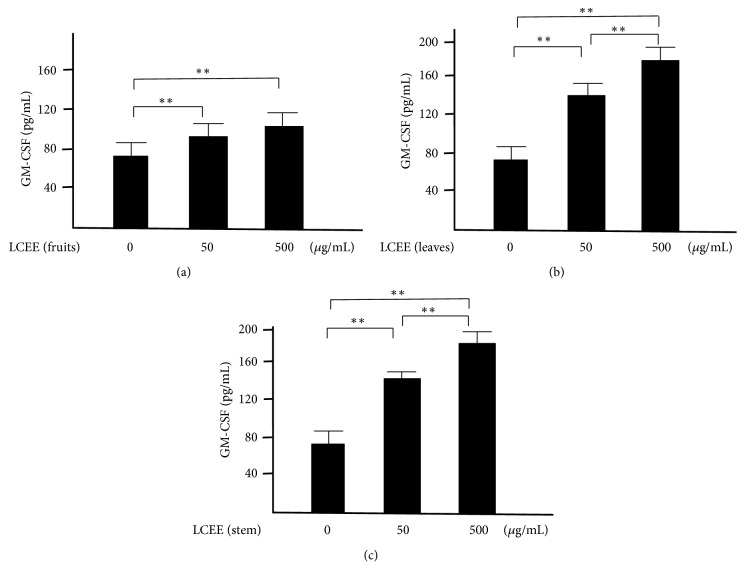
GM-CSF levels in culture medium of mesenteric lymph nodal cells. LCEEs ((a) fruits, (b) stems, and (c) leaves) were added to cells. GM-CSF was measured by ELISA. Data represent the mean ± SD (*n* = 6). *∗∗p*< 0.01 by Bonferroni-Dunnett's* t*-test.

Next, splenocytes and mesenteric lymph nodes cells isolated from ICR mice were incubated with each LCEEs for 24 hours, respectively. The concentrations of GM-CSF in the culture solution were measured. As a result, each LCEEs showed significant differentiated effect as dose-dependent manner in both splenocytes (Figure 9) and mesenteric lymph nodes cells (Figure 10). The activities of LCEEs from leaves and stems seemed to be higher than that of LCEEs from fruits.

### 3.5. Evaluation of Proliferative Response of Splenocytes and Mesenteric Lymph Nodal Cells without LPS

**Figure 11:**
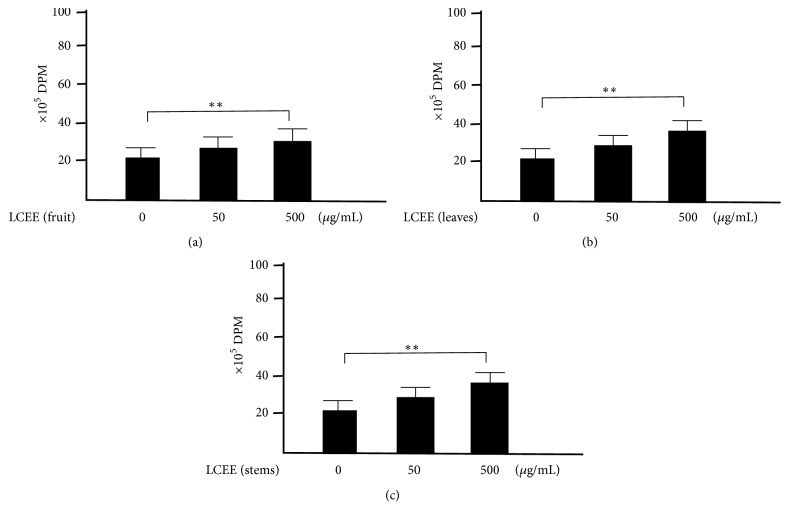
^3^H-thymidine-uptake assay in splenocytes without LPS stimulation. LCEEs ((a) fruits, (b) stems, and (c) leaves) were added to cells. Data represent the mean ± SD (*n* = 6). *∗∗p*< 0.01 by Bonferroni-Dunnett's* t*-test.

**Figure 12:**
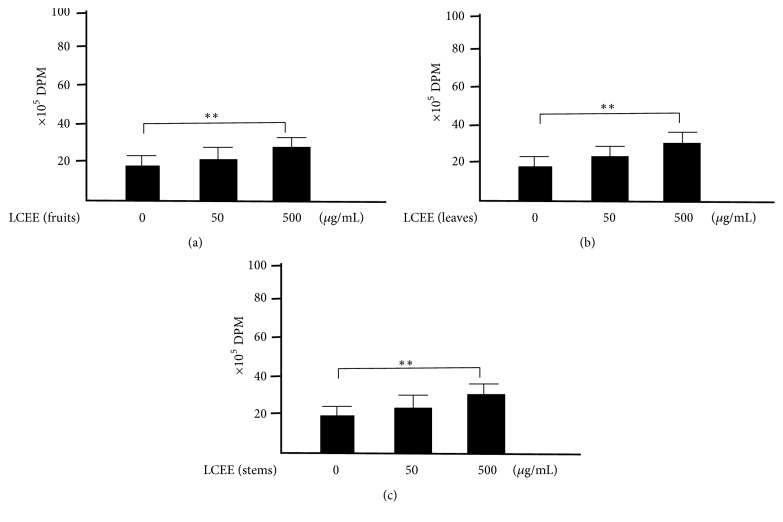
^3^H-thymidine-uptake assay in mesenteric lymph nodal cells without LPS stimulation. LCEEs ((a) fruits, (b) stems, and (c) leaves) were added to cells. Data represent the mean ± SD (*n* = 6). *∗∗p*< 0.01 by Bonferroni-Dunnett's* t*-test.

We also tried to clarify whether splenocytes treated with LCEEs showed elevated activities without LPS, we performed ^3^H-thymidine uptake analysis. As shown in Figure 11, the uptake of ^3^H-thymidine into splenocytes treated with LCEEs (500 *μ*g/mL) was significantly higher than that of control (0 *μ*g/mL). We also confirmed that the uptakes of ^3^H-thymidine into mesenteric lymph nodal cells treated with LCEEs (500 *μ*g/mL) exhibited significantly higher activity than those without the treatments (Figure 12).

### 3.6. Lignin Contents

**Figure 13:**
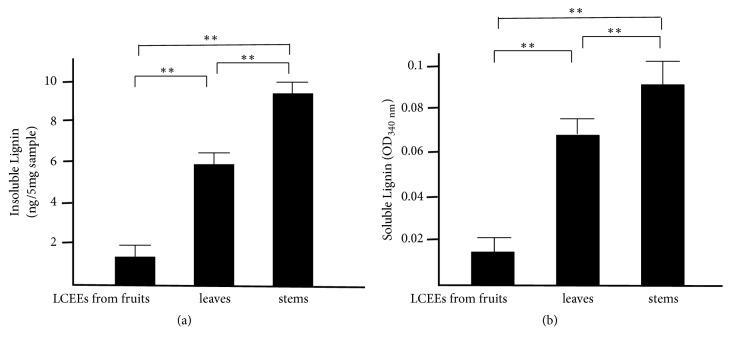
Lignin concentration in some parts of LCEEs. Insoluble lignins were measured as weight. Soluble lignins were measured as optical density (OD_340 nm_). Data represent the mean ± SD (*n* = 6). *∗∗p*< 0.01 by Bonferroni-Dunnett's* t*-test.

The concentrations of acid insoluble and soluble lignins in LCEE prepared from leaves were significantly higher than that in LCEEs from the fruits, and that from the stems was significantly higher than that in LCEEs from the leaves (Figure 13). Thus, the concentrations of total lignins in stem were the highest among three parts of LCE.

## 4. Discussion

To our knowledge, this is the first experimental study that leaves and stems of the LCE would be effective in* S. pyogenes*-caused murine model. Our result revealed that LCEE prepared from the leaves and stems showed a stronger immunostimulative action than that from fruits. As leaves and stems are unused resources, they will be potentially useful materials that can be expected for future medical applications.

It is not surprising that the leaves and stems of the LCE are used as a crude drug because an herbal medicine having a detoxifying action. Especially, some physiological activity is recognized on the stems of the* Lonicera japonica* [14]. We first evaluated the possibilities of some parts of LCE as anti-infective materials in animal study. From these results, we next investigated the immunostimulatory effects of LCEEs by the inducible effects on GM-CSF secretion from lymph nodes. The maximum concentration of LCEE* in vitro* study was set at 500 *μ*g/ml because this concentration refers to the fact that 4% of the extract was absorbed and distributed in blood when human being takes 50 g of the extract. From our experimental results, the extraction efficiency from the fresh fruits of LCE was 12.6%. Let us assume that a human eats 100 g of fruits of LCE (whose amount of LCE extract is 12.6 g). If this is all absorbed and evenly distributed in the blood, the blood concentration of the LCE extract may be 2.7 mg/mL because the human blood volume is about 4.6 L. In fact, however, not all of the LCEE can be absorbed into the body. Now we do not know the exact absorption rate of the LCEEs, but if it is about 10% similar to the iron absorption rate [22], the blood concentration of the LCE extracts may become 270 *μ*g/mL. Since this hypothesized blood concentration is included between 50 to 500*μ*g/mL of this setting concentration, we think that our concentration setting of LCEEs is reasonable in this study. There is no report on the improvement of GALT-related immune mechanism by* Lonicera* genus including in LCE. However, in other plant investigation, murine GALT function was improved via improvement of Th2 cytokine IL-4 level when cranberry proanthocyanidin was administered to low GALT function-mouse [23]. Furthermore, in traditional Japanese Kampo medicine and traditional Chinese medicine, both of which are originated from ancient Chinese medicine, the stimulation of lymphocytes derived from spleen, mesenteric lymph node, and Peyer's patch of mice orally administered with Juzen-Taiho-To with concanavalin A causing the production of IFN-*γ* was enhanced in spleen, mesenteric lymph node, and Peyer's patch-derived lymphocytes [24]. As LCEEs may also have same mode of action for improving GALT function as cranberries and Juzen-Taiho-To, further research is necessary for this point. G-CSF and GM-CSF are cytokines that stimulate the production of granulocytes and are clinically used to treat neutropenia and to prevent immune deficiency induced by chemotherapy [25, 26]. In other cases, the extract of hochuekkito, one of the immunostimulatory agents in Japanese traditional Kampo formulation, stimulated G-CSF secretion from intestinal epithelial cells, and its active ingredients were polysaccharides [27]. Regarding LCEE, the same kind of mode of action may be suggested.

Although the various parts of* Lonicera* species have been utilized in folk medicine for many decades, several phenolic matrix constituents have been suggested as the main components responsible for the health benefits of the edible honeysuckle recently [4]. The phenolic fraction of LCE fruits may be beneficial for the adjunctive treatment of periodontitis as an agent for attenuation of the inflammatory process [28]. It also inhibited LPS-induced upregulation of interleukin-1*β* and interleukin-6 in gingival fibroblast and it suppressed expression of cyclooxygenase-2 [29]. This immunological result was almost coincided with our result. An LCEEs also showed endotoxin-induced uveitis. The possible mechanism for these effects may depend especially on the ability to inhibit activation of NF-*κ*B and the subsequent production of proinflammatory mediators such as TNF-*α*. As murine macrophage cell lines were stimulated with LPS in the presence of blue honeysuckle extract, the treatment with this extract significantly reduced the inflammatory cell migration and the levels of TNF-*α*.

Our results demonstrated that the stems and leaves of LCE had more immunological activity than the fruit. We focused on lignins since lignin-carbohydrate complexes (LCCs) are major cell wall components formed by the dehydrogenation of three monolignols,* p*-coumaryl, coniferyl, and sinapyl alcohols. LCCs stimulated the iodination of myeloperoxidase-positive human monocytes, neutrophils, and promyelocytic leukemia that may be involved in the bacterial killing mechanism. LCCs stimulated splenocyte proliferation and showed both pro- and anti-inflammatory activity in activated macrophage. Preliminary DNA array analysis demonstrated the activation of the signal pathway of chemokine expression via Toll-like receptor 2. Broad and potent antiviral activity and synergism with vitamin C suggested functionality of LCCs as alternative medicine [30]. Various lignified materials, including pine cone extract, stimulated the morphological change of mouse peritoneal macrophages. The results strongly suggest the importance of lignin-structure in macrophage activation [31].

The water-soluble lignin in the extract of the solid culture medium of* Lentinus edodes mycelia* has been known to have immunopotentiating activities* in vivo* and* in vitro*. It activated the cytotoxicity of natural killer cells and macrophages and activated T cells in vitro. It also had antiviral and immunopotentiating activities [32]. Since there has been no report on the analysis of the immunological effect on lignins in LCE, future research is strongly desired from the viewpoint of searching for new drugs.

In summary, LCEEs prepared from the fruits, leaves, and stems may improve the immunological effect on immunocompromised condition. Furthermore, LCEEs from the leaves and stems had more effective than that from the fruit. We suggest LCEE as the therapeutic candidate for novel effective therapy on bacterial infectious disease caused by* S. pyogenes*.

## Data Availability

The data used to support the findings of this study are available from the corresponding author upon request.
